# An Evolutionary Firefly Algorithm for the Estimation of Nonlinear Biological Model Parameters

**DOI:** 10.1371/journal.pone.0056310

**Published:** 2013-03-04

**Authors:** Afnizanfaizal Abdullah, Safaai Deris, Sohail Anwar, Satya N. V. Arjunan

**Affiliations:** 1 Artificial Intelligence and Bioinformatics Group, Faculty of Computing, Universiti Teknologi Malaysia, Johor, Malaysia; 2 Pennsylvania State University, Altoona, Pennsylvania, United States of America; 3 Laboratory for Biochemical Simulation, RIKEN Quantitative Biology Center, Osaka, Japan; West Virginia University, United States of America

## Abstract

The development of accurate computational models of biological processes is fundamental to computational systems biology. These models are usually represented by mathematical expressions that rely heavily on the system parameters. The measurement of these parameters is often difficult. Therefore, they are commonly estimated by fitting the predicted model to the experimental data using optimization methods. The complexity and nonlinearity of the biological processes pose a significant challenge, however, to the development of accurate and fast optimization methods. We introduce a new hybrid optimization method incorporating the Firefly Algorithm and the evolutionary operation of the Differential Evolution method. The proposed method improves solutions by neighbourhood search using evolutionary procedures. Testing our method on models for the arginine catabolism and the negative feedback loop of the p53 signalling pathway, we found that it estimated the parameters with high accuracy and within a reasonable computation time compared to well-known approaches, including Particle Swarm Optimization, Nelder-Mead, and Firefly Algorithm. We have also verified the reliability of the parameters estimated by the method using an *a posteriori* practical identifiability test.

## Introduction

The elucidation of the dynamic behaviour of biological processes that are made up of complex networks is a key topic in systems biology. Mathematical models are popular for such studies, because they can test predictions and generate hypotheses for experimental analyses about the processes. These models are constructed with time derivative expressions, such as ordinary differential equations (ODEs), to describe the change of certain quantities of interest over time [1].

Normally, models consist of a set of parameters that represent the physical properties of the system, such as biochemical reaction rates. Measurement of these parameters is often difficult and in some cases impossible [2]. Parameters are usually estimated by fitting the predicted data from a model to experimental time-series measurements. The fitting, which is performed by minimizing the error between the two sets of data by adjusting the model parameters, is an optimization problem. Local optimization methods [3] such as Levenberg-Marquardt [4], gradient descent [5], Nelder-Mead [6] and least-squared fitting [7] have been extensively utilized for this purpose. These methods exploit a given set of initial values within a specified search space to find optimal parameter values, which correspond to the local minimum error between the experimentally measured and predicted data. However, it is difficult to find a global minimum when the initial values are not carefully selected. Furthermore, the measured data usually suffer from noise and experimental errors [8], [9], impairing accurate solutions. To handle noisy data, statistical based methods have received considerable attention [1], [8]. Methods such as maximum-likelihood [10] and Bayesian inference [11] employ probabilistic based approaches to infer the parameters. However, these methods incur significant computational cost, especially when solving high dimensional parameter estimation problems, and require intricate derivatives that demand large constraint adjustments.

Recently, global optimization methods have also gained much focus [9], [12], [13], [14]. These methods employ stochastic searching techniques for a set of potential solutions that are randomly selected within a given search space. Particle Swarm Optimization (PSO) [15], [16], Genetic Algorithms (GA) [17], [18], Simulated Annealing [19], [20], Scatter Search (SS) [21], [22], and Differential Evolution (DE) [23] have already been used to estimate the parameters of various biological models [9], [24], [25], [26]. The main advantage of these methods is their ability to find global optimum solutions in nonlinear and high dimensional problems. In addition, they are generally derivative-free and are easy to implement. However, since they look for a global optimum solution over the entire search space, a significant amount of computation time is required [9], [24].

Various optimization methods have been hybridized to capture the best features of each while reducing the computational cost [12]. Balsa-Canto and co-workers [25] presented a general strategy to switch between global and local searching techniques, showing this to be effective at estimating the parameters of biological systems. More recently, Fu and co-workers [27] proposed a hybrid method that improved the conventional velocity updating strategy in PSO by incorporating the evolutionary operations of the DE method. Ho and Chan [26] employed the local Taguchi method to enhance evolutionary operations of DE and applied it to estimate the parameters of a HIV model. We have recently reported a preliminary effort [28] to hybridize the Firefly Algorithm (FA) [29] with the evolutionary operations of the DE method. Among these methods, evolutionary computation was shown to enhance accuracy and reduce computation time.

To date, hybridization works have not focused on the issue of model identifiability [30], [31], which is important when developing predictive biological models [32]. A non-identifiable model has no unique values for the parameters and, as a result, similar model predictions can be obtained from different parameter values. Statistically, a model is non-identifiable if it has two different parameter values that produce the same probability distribution of the observable variables. On the other hand, a model is identifiable if its true parameter values can be determined from a sufficient number of observation data. Identifiability can be classified as structural [32], [33] or practical [34], [35]. The model structure, which depends on the dynamics of the system and the conditions of the stimuli and observation, determines structural identifiability, whereas practical identifiability relies on the completeness of the sampling data and the lack of measurement noise.

Identifiability is important in biological models since we can only make valid inferences from models that are at least partially identifiable. In addition, optimization methods cannot estimate parameters reliably if the model is structurally non-identifiable. As such, in this work we focus on developing an optimization method for structurally identifiable models. However, to ensure that the estimated parameters are reliable and thereby are a unique solution to the particular model, we perform a practical identifiability test after the estimation procedure.

Here, we extend FA to estimate the parameters of nonlinear biological models. The FA method employs a population-based iterative procedure with a number of agents that synchronously solve an optimization problem [36]. Since it adopts stochastic searching techniques similar to PSO and GA, a substantial amount of computation time is required to obtain good estimation accuracies. We attempt to address the computation time issue by incorporating an evolutionary operation from the DE method [12], specifically by relocating the agents in each subsequent iteration. Compared to our preliminary work [28], the present study introduces a discrimination step that classifies the solutions into two sub-groups: potential and weak solutions, and ensures that the computation time is utilized more efficiently by preserving those solutions with favorable fitness in each iteration. The proposed method is tested to estimate the parameters of models for the p53 signalling pathway negative feedback loop [37] and arginine catabolism [38]. We compare the results from the proposed method and the Nelder-Mead, PSO, and FA methods. In addition to exhibiting improved accuracy and convergence speed, the method also showed that it is reliable in estimating parameters in a practical identifiability test [1], [8].

## Methods

In this section, we begin with the problem formulation by mathematically representing a target biological process and the parameter estimation problem, followed by a detailed description of the proposed optimization method and the identifiability test. The test verifies the reliability of the estimated parameter and is performed *a posteriori*, i.e., after conducting the estimation procedure.

### Problem Formulation

A biological process can be represented as a series of ordinary differential equations (ODEs) in the following form:
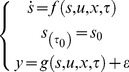
(1)where *s* is the state vector, which depicts the concentration of a molecule species; *u* is the input signal to the process, such as temperature changes; *x*  =  {*x*_1_, *x*_2_, *x*_3_,…, *x*_M_} denotes the parameters such as kinetic rates; *τ* is the sampling time; and *y* represents the measurable data points or output variables [1], [8]. In general, experimental data exhibit measurement noise. To represent this property in the formulation, the output function, *g* is superimposed with the uncorrelated Gaussian noise, *ε* [39], [40].

**Figure 1:**
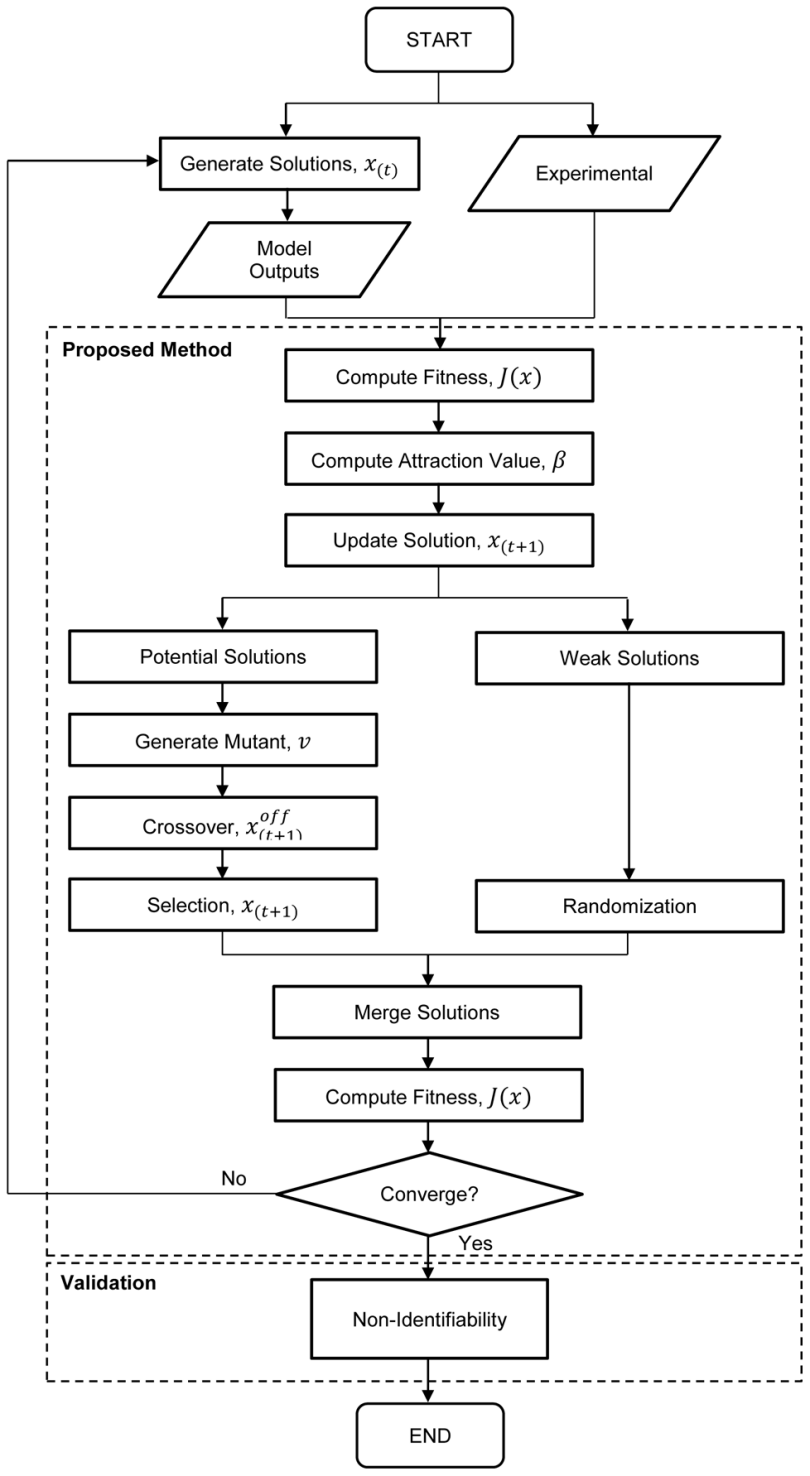
Flowchart of the parameter estimation problem. The parameter estimation procedure begins with a prediction from the model and reference data obtained from experiments. The predictions are generated from an ODE solver. The difference between the predicted and the expected data is computed in an iteration. The iteration is repeated until an optimal parameter set is found by minimizing the difference.

The parameter estimation problem can be categorized as an optimization problem since it aims to find the optimal values of the parameter set, *x*, such that the difference between the experimental data, 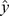, and the state vector produced by the model, 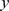, is minimized. The optimization problem can be expressed by the following nonlinear least squared function:
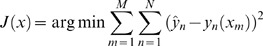
(2)where *x* is the solution representing the set of parameters; *M* and *N* are the total number of parameters to be estimated and sampling times, respectively [39]. Estimating the parameters is nontrivial because of the nonlinearity of the problem can result in suboptimal values. Figure 1 illustrates the parameter estimation framework. It consists of the optimization method that searches for suitable parameter values and the ODE solver that generates model predictions.

### An Evolutionary Firefly Algorithm

We propose an improvement to FA by introducing an evolutionary operation to the selected fireflies in a population. Each *i*th firefly denotes a vector, *x_i_*  =  {*x_i_*_1_, *x_i_*_2_, *x_i_*_3_,…, *x_iM_*}, that represents a set of *M* parameters of the model. The population of fireflies is initialized randomly. The position of each firefly is constrained to not exceed the range of the search space by
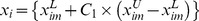
(3)where *x_i_* is the vector of the *i*th solution, *C*_1_ is a uniformly distributed random value between 0 to 1, 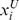
and 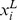 are the predefined upper and lower bounds, respectively, and 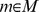 [23]. Once the vector is determined, the fitness value of each *i*th solution, *J*(*x_i_*), is calculated. The fitness of each solution is compared with its neighbours. If a neighbour is fitter, the distance between the pair of *i*th and *j*th solutions is computed and the attraction value, 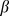
is calculated as follows
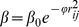
(4)where 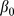 is the initial attractiveness, e is the standard exponential constant, *φ*, is the predefined light absorption coefficient, and r_ij_ is the Euclidean distance between the *i*th and *j*th solutions [29]. The vector update is only performed when the fitness of the neighbouring *j*th solution is better than the current *i*th solution using the expression
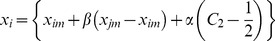
(5)where α and *C*_2_ are uniformly distributed random numbers in the range 0 to 1. Thus, the updates allow the solutions to move towards that with the current optimal fitness and utilize the search space more efficiently [29], [36]. The iteration is repeated until all solutions have been updated. The solution that produces the best fitness is selected as the global best solution. The population, now containing updated solutions, is then sorted according to the fitness into two parts: potential solutions, which consists of the fittest solutions, and weak solutions, which contains the remainder. The solution vectors are updated according to
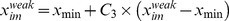
(5)where *x*_min_ is the vector of current best solution and *C*_3_ is a uniformly distributed random number between 0 and 1 [23].

**Figure 2:**
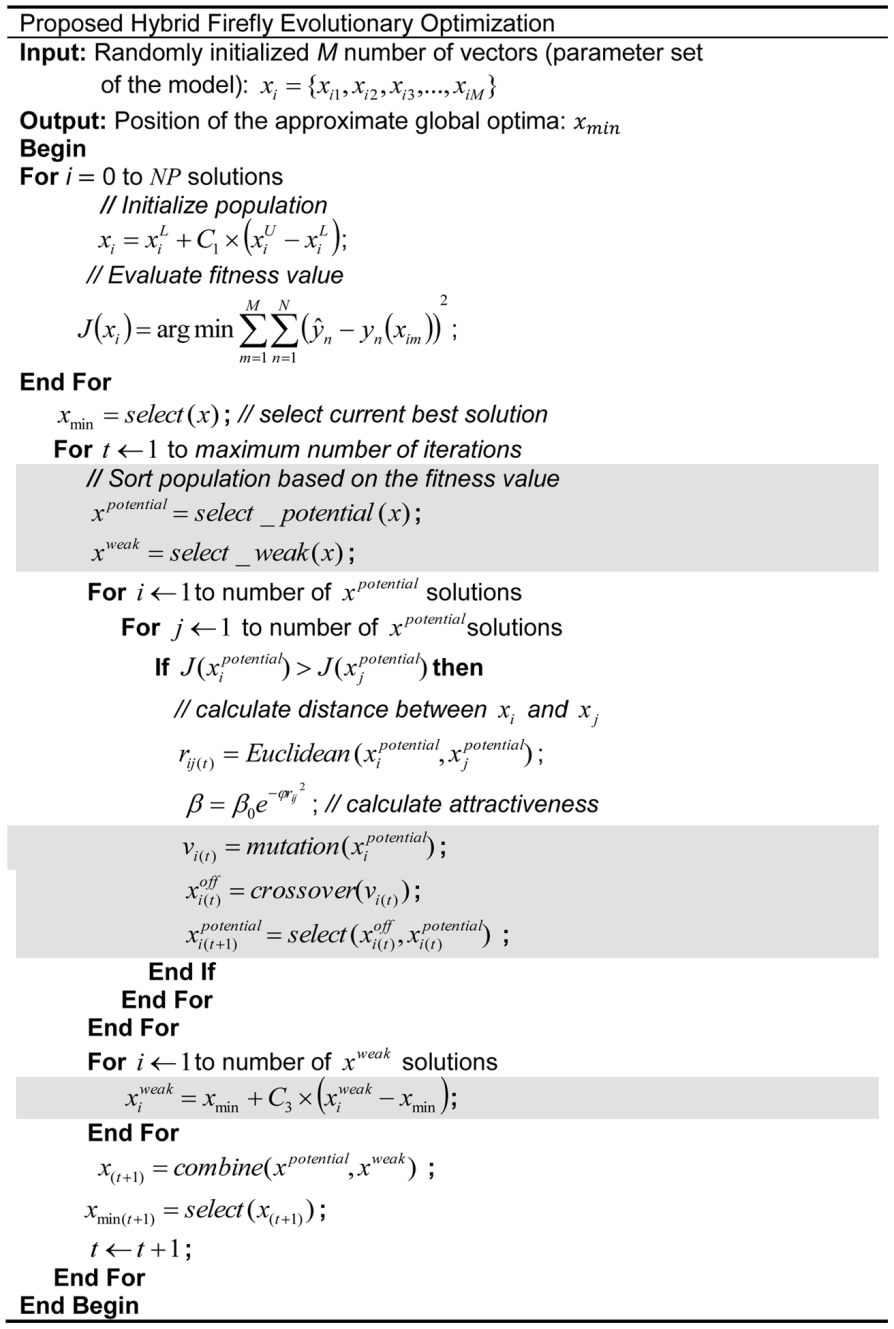
Algorithm of the proposed method. The proposed method is composed of the two major steps indicated by the shaded sections. The first step sorts the population according to fitness into two sub-populations: potential and weak. The potential sub-population is subjected to evolutionary improvements. In the last step, a random vector update is performed on the solutions within the weak sub-population.

Evolutionary operations are performed simultaneously on the potential solutions. First, a mutation step is executed for each solution,
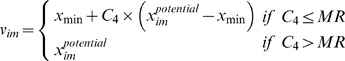
(6)where *v_i_* is the mutated solution vectors, *C*_4_, is a uniformly distributed random number between 0 and 1, and *MR* is a predefined mutation rate [23]. A new breed of solutions is then created by a crossover step according to the condition
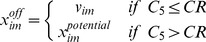
(7)where *x^off^_i_* is the vector of offspring solutions, *C*_4_ is a uniformly distributed random number between 0 and 1, and *CR* is a predefined crossover rate [23]. The fitness of each offspring solution is calculated and, to retain the population size, a simple selection is done according to [23]



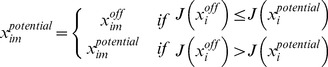
(8)These solutions are then inserted into the original population. The solution that yields the best fitness within the population is set as the current best firefly and the value is noted as the current global optimum. This procedure is repeated until the maximum number of iterations is reached or an acceptable fitness value is found. The overall procedure of the proposed method is depicted in Figure 2.

### Identifiability Test

We perform an identifiability test based on the simple approximation of the variance of random noise variables [1], [8]. Consider a set of time series data that is measured at discrete time intervals, with the model expressed as
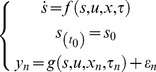
(9)where *n* stipulates the number of samples. Assume that by executing the optimization procedure, an estimated parameter, 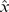
is found in which 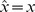. Thus, the measurement noise of the component can be written as [1], [8]



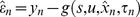
(10)If 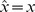, and accordingly 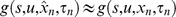, the variance of 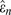 given by 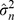 will be nearer to the real variance of *ε_n_* Thus, 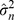
can be estimated from
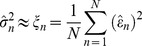
(11)where *N* is the total number of samples. Consequently, the interval of the variance can also be estimated from the confidence level, *γ*  =  1–*δ*, and 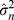
will lie within the interval 
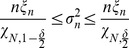
(12)


**Table 3:** χ2 test for the parameter estimation of the p53 negative feedback loop model using the proposed method.

	*A*	*B*	*C*	*D*
Real Variance (*σ_k_^2^*)	3.53×10^−1^	3.24×10^−1^	3.30×10^−1^	3.28×10^−1^
Variance Point (*ξ_k_*)	3.52×10^−1^	3.24×10^−1^	3.31×10^−1^	3.29×10^−1^
Variance Interval	[3.28×10^−1^, 3.79×10^−1^]	[3.02×10^−1^, 3.49×10^−1^]	[3.08×10^−1^, 3.56×10^−1^]	[3.06×10^−1^, 3.55×10^−1^]
χ^2^ Test	**Pass**			

with a probability of 100*γ*%. Here, χ*_N,δ_* represents the 100*δ*th percentile of the χ^2^ distribution with *N* degrees of freedom [1], [8]. If the actual variance, 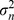, does not lie within the interval, the measurements, *y_n_*, could not have been produced by the parameter 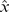. Hence, 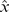 is considered inaccurate with a confidence of 100*γ*% [1], [8]. In this work, we set the value of *γ* to 0.95, and thus fixed the significance level to 0.05.

**Table 4:** χ^2^ test for the parameter estimation of the p53 negative feedback loop model using the Nelder-Mead method.

	*A*	*B*	*C*	*D*
Real Variance (*σ_k_^2^*)	3.53×10^−1^	3.24×10^−1^	3.30×10^−1^	3.28×10^−1^
Variance Point (*ξ_k_*)	5.07×10^−4^	2.55×10^−4^	689×10^−4^	8.21×10^−5^
Variance Interval	[4.71×10^−4^, 5.46×10^−4^]	[2.37×10^−4^, 2.75×10^−4^]	[6.41×10^−4^, 7.42×10^−4^]	[7.64×10^−5^, 8.86×10^−5^]
χ^2^ Test	Fail			

### Model Selection

Model selection is a crucial step in biological system modelling since many variations of models are available with different experimental conditions and assumptions [1], [8], [41]. Here, we perform a model selection procedure to evaluate the relevancy of the experimental conditions and assumptions to fit a given set of measurements. We used two approaches to select the model that best fits the data. The first approach employs the measurement error variance points and intervals [1], [8]. Consider two distinct models that are represented in the form of (1) and are rewritten as
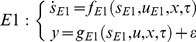
(13)

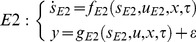
(14)


**Table 5:** χ^2^ for the parameter estimation of the p53 negative feedback loop model using the PSO method.

	*A*	*B*	*C*	*D*
Real Variance (*σ_k_^2^*)	3.53×10^−1^	3.24×10^−1^	3.30×10^−1^	3.28×10^−1^
Variance Point (*ξ_k_*)	5.88×10^−4^	2.03×10^−4^	7.99×10^−4^	2.22×10^−4^
Variance Interval	[5.48×10^−4^, 6.34×10^−4^]	[1.89×10^−4^, 2.19×10^−4^]	[7.44×10^−4^, 8.61×10^−4^]	[2.07×10^−4^, 2.39×10^−4^]
χ^2^ Test	Fail			

From the above expressions we know that the same experimental data is used in both models. The variance point and intervals of the models can therefore be computed following the procedure described in the previous section.

**Table 6:** χ^2^ test for the parameter estimation of the p53 negative feedback loop model using the FA method.

	*A*	*B*	*C*	*D*
Real Variance (*σ_k_^2^*)	3.53×10^−1^	3.24×10^−1^	3.30×10^−1^	3.28×10^−1^
Variance Point (*ξ_k_*)	1.01×10^−5^	7.12×10^−6^	6.66×10^−5^	2.27×10^−6^
Variance Interval	[1.00×10^−5^, 1.16×10^−5^]	[6.62×10^−6^, 7.67×10^−6^]	[8.05×10^−5^, 9.32×10^−5^]	[1.55×10^−4^, 1.79×10^−4^]
χ^2^ Test	Fail			

In the second approach [41], the Akaike Information Criterion (AIC) [42], [43] is used. The measurement noise is assumed to be independent and normally distributed. We use the following expression of the AIC [41],
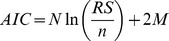
(15)where *N*, *RS*, and *M* are the number of samples, the residual sum of squares and the number of parameters, respectively. A model that yields a smaller AIC value is considered as the better model [41].

## Results

To evaluate the performance of the proposed method, we estimated the parameters of models for the negative feedback loop of p53 signalling pathway and arginine catabolism. The models contain both small and large number of parameters with noisy and incomplete measurements. The experimental data for both models were generated *in silico* [1] and added with 25% white Gaussian noise. The reliability of the estimated parameters is verified by the practical identifiability test, as previously performed by Lillacci and Khammash [1], [8].

### The Negative Feedback Loop of p53 Signaling Pathway

p53 is a tumour-suppressor protein that regulates the activity of hundreds of genes involved in cell growth and death [44], [45]. It also plays a crucial role in preventing cancer [46]. The accumulation and activation of p53 is controlled by several stress signals, including DNA damage, hypoxia, heat shock, nutrient deprivation and oncogene activation [37]. p53-regulated genes produce proteins that communicate the stress signals to adjacent cells and constitute feedback loops that increase or reduce p53 activity [44]. A p53 negative regulator, Mdm2 has been suggested to be an important factor in oncogene activation [37]. It is an E3 ligase that ubiquitinates p53 by direct association and inhibits its transcriptional activity [37]. Simultaneously, p53 also regulates the mdm2 gene, resulting in a negative feedback loop [47].

Recently, Hunziker, Jensen and Sandeep [37] developed a model of the p53-Mdm2 feedback loop to investigate the integration of multiple stress signals. The model can be used to predict the stress signal that produces a high p53 response and is represented as [37]

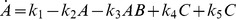
(16)

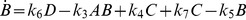
(17)

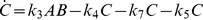
(18)

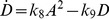
(19)where *A*, *B*, *C*, and *D* are nuclear-p53, Mdm2, the p53-Mdm2 complex, and Mdm2 mRNA, respectively. The parameters *k*_1_, *k*_2_, *k*_3_, *k*_4_, *k*_5_, *k*_6_, *k*_7_, *k*_8_, and, *k*_9_ are the rates of p53 production, p53 degradation, p53-Mdm2 complex formation, p53-Mdm2 complex diffusion, Mdm2 degradation, Mdm2 translation, Mdm2-mediated degradation of p53, Mdm2 transcription, and Mdm2 mRNA degradation, respectively [37]. To evaluate the robustness of the proposed method, it was used to estimate all nine parameters of the model with incomplete and noisy experimental data.

The solution vector for the optimization problem can be given as *x_i_*  =  {*k*_1_, *k*_2_, *k*_3_, *k*_4_, *k*_5_, *k*_6_, *k*_7_, *k*_8_, *k*_9_} for *i*  =  {1,2,3,…,*NP*}, where *NP* is the size of the solution. We evaluated the performance of our method against the FA, Nelder-Mead, and PSO methods. Each method is subjected to a set of 100, 200, and 500 iterations with 20, 40, 80, and 100 solutions. The average and the standard deviation of the best fitness values, which are calculated out of 100 runs, are listed in Table 1. The results indicate that the proposed method is able to find better fitness values with smaller deviations than the other tested methods.

**Table 1:** Average best fitness and standard deviation (presented within bracket) for the p53 negative feedback loop model.

No. of Solutions	No. of Iterations	Nelder-Mead	PSO	FA	Proposed
20	100	9.64×10^−4^ (3.22×10^−4^)	3.19×10^−4^ (1.31×10^−4^)	1.55×10^−5^ (1.01×10^−5^)	1.34×10^−7^ (1.15×10^−7^)
	200	3.17×10^−4^ (2.92×10^−4^)	2.09×10^−4^ (2.15×10^−4^)	9.89×10^−6^ (5.51×10^−6^)	8.55×10^−8^ (3.96×10^−8^)
	500	3.02×10^−4^ (3.03×10^−4^)	1.98×10^−4^ (1.01×10^−4^)	8.21×10^−6^ (4.54×10^−6^)	1.25×10^−8^ (2.50×10^−8^)
40	100	7.20×10^−5^ (5.02×10^−5^)	4.04×10^−5^ (3.13×10^−5^)	2.20×10^−6^ (2.11×10^−6^)	1.56×10^−8^ (2.28×10^−8^)
	200	5.25×10^−5^ (2.12×10^−5^)	2.59×10^−5^ (1.98×10^−5^)	8.22×10^−7^ (2.01×10^−7^)	7.96×10^−9^ (1.28×10^−9^)
	500	2.23×10^−5^ (1.92×10^−5^)	1.79×10^−5^ (1.51×10^−5^)	3.65×10^−7^ (2.71×10^−7^)	2.16×10^−9^ (1.08×10^−9^)
60	100	7.04×10^−5^ (5.05×10^−5^)	2.15×10^−5^ (2.01×10^−5^)	1.12×10^−6^ (1.10×10^−6^)	9.25×10^−9^ (2.52×10^−9^)
	200	5.51×10^−5^ (3.35×10^−5^)	9.35×10^−6^ (1.11×10^−6^)	8.75×10^−7^ (5.60×10^−7^)	3.28×10^−9^ (1.02×10^−9^)
	500	3.27×10^−5^ (1.50×10^−5^)	7.05×10^−6^ (5.81×10^−6^)	5.05×10^−7^ (5.21×10^−7^)	9.95×10^−10^ (4.18×10^−10^)
80	100	5.23×10^−6^ (3.36×10^−6^)	1.77×10^−6^ (1.35×10^−6^)	1.04×10^−7^ (1.78×10^−7^)	3.21×10^−9^ (1.02×10^−9^)
	200	3.02×10^−6^ (2.96×10^−6^)	9.27×10^−7^ (5.15×10^−7^)	8.84×10^−8^ (2.80×10^−8^)	9.58×10^−10^ (5.52×10^−10^)
	500	2.95×10^−6^ (1.06×10^−6^)	7.57×10^−7^ (3.19×10^−7^)	5.50×10^−8^ (3.25×10^−8^)	5.01×10^−10^ (4.89×10^−10^)

**Figure 3:**
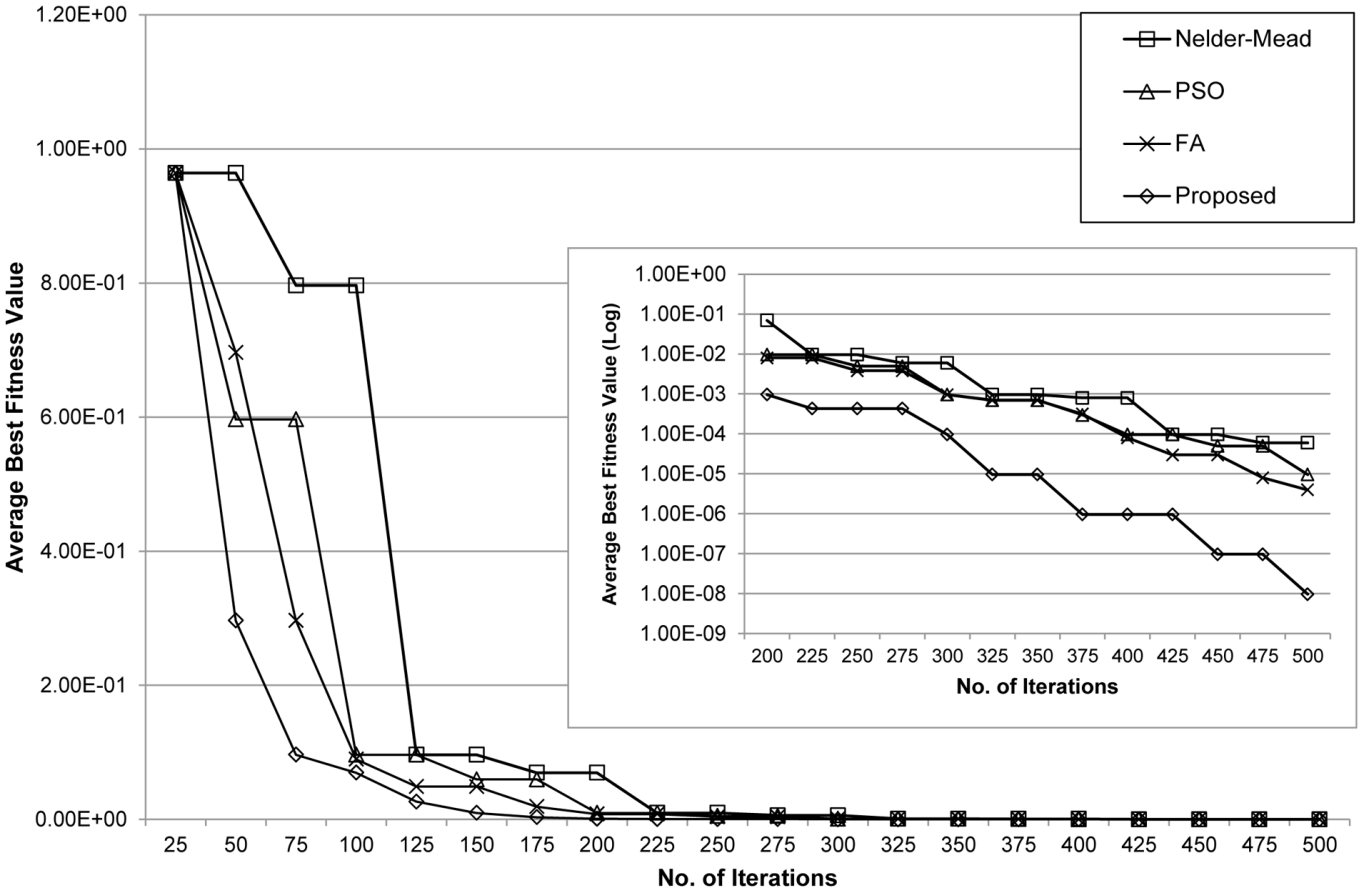
Convergence behaviour for the p53 negative feedback loop model. Plots show the average best fitness values of the Nelder-Mead, PSO, FA and proposed methods at each iteration.

Figure 3 compares the fitness convergence of the evaluated methods. Overall, the proposed method exhibited improved convergence times and escaped suboptimal solutions compared to those produced by the other methods. The local Nelder-Mead method converged to suboptimal solutions several times and ended at one of them when the maximum number of iterations is reached. Consequently, this method may be suitable for problems having a small number of parameters, but in high dimensional complex problems it would likely generate unacceptable results. Although the PSO method can escape suboptimal solutions, it suffers from longer computation times.

All computations were performed on the same 64-bit platform, powered by an Intel Core i5 1.5 GHz central processing unit (CPU) with 4 GB of memory. Table 2 lists the average amount of computation time of each method for 500 iterations in 100 independent runs. Generally, the results show that the proposed method requires a computation time that is less than PSO and FA and similar to the Nelder-Mead method. The reduced time is the result of the improved searching strategy adopted by the proposed method.

**Table 2:** Average computation times, in second, for the p53 negative feedback loop model. Average time taken for 500 iterations in 100 independent runs.

No. of Solutions	Average Computation Times (s)			
	Nelder-Mead	PSO	FA	Proposed
20	33.5	45.3	40.5	36.8
40	52.9	61.0	58.2	53.3
60	71.2	80.3	75.6	73.0
80	95.6	112.4	106.1	98.4

**Figure 4:**
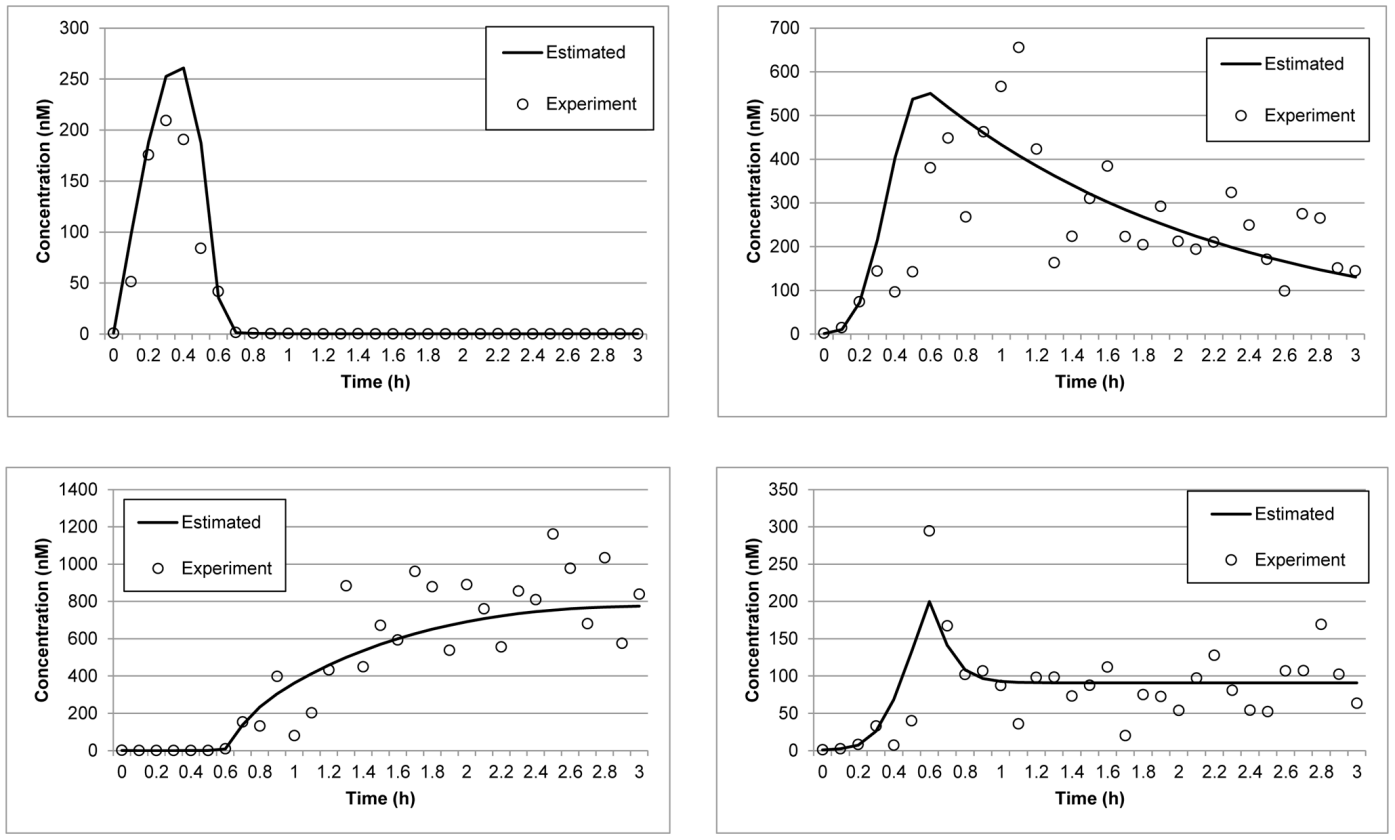
Data fitting for the p53 negative feedback loop model. Data points (circles) represent synthetic measurements obtained by adding Gaussian noise to the model prediction (crosses). Lines represent the reconstructed model using the parameters estimated by the proposed method. The upper left and right panels illustrate the concentrations of nuclear-p53 and Mdm2, respectively. The lower left and right panels represent the concentrations of p53-Mdm2 and Mdm2 mRNA, respectively.

The real variances arising from the noisy experimental data were computed as 3.53×10^−1^, 3.24×10^−1^, 3.30×10^−1^, and 3.28×10^−1^ for *A*, *B*, *C* and *D*, respectively. Table 3 to 6 list the variance points and the corresponding intervals for each method. The variance points of the proposed method agree with the real variances and lie within the expected variance intervals, whereas those of the other methods do not. Furthermore, the variance points of the other methods are also significantly smaller than the real variances and have larger intervals than the proposed method. Generally, variance points that are small and within the expected intervals indicate that the model output is reliable and consistent with the data set. However, if the points are smaller than the expected values, as described above, it implies that the data has been overfitted when estimating the parameters. Overfitting can be caused by insufficient experimental data or, as in the case here, susceptibility to noise in the data when estimating a large number of parameters. As such, although the errors between the model outputs of the other methods and the data are smaller because of overfitting, the models themselves do not accurately represent the system and would generate erroneous outputs. Tables 3, 4, 5, 6 also show that the Nelder-Mead, PSO, and FA methods have failed the χ^2^ test with a confidence level of 95%. Together, the results demonstrate that the proposed method is more robust to the measurement noise since it has passed the χ^2^ test and has good variance points within the expected intervals. Figure 4 illustrates the data fit of the reconstructed model using the parameters of the proposed method and the corresponding experimental data. The figure clearly shows that the results from the estimates are consistent with the curves obtained from the experiments.

Table 7 compares the model selection results of the original model (Eq. 16–19) with a modified form represented by the following equations:
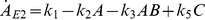
(20)

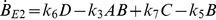
(21)

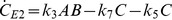
(22)

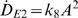
(23)


**Table 7:** p53 negative feedback loop model selection results.

		*A*	*B*	*C*	*D*
Real Variance (*σ_k_^2^*)		3.53×10^−1^	3.24×10^−1^	3.30×10^−1^	3.28×10^−1^
E1	Point (*ξ_k_*)	3.52×10^−1^	3.24×10^−1^	3.31×10^−1^	3.29×10^−1^
	Interval	[3.28×10^−1^, 3.79×10^−1^]	[3.02×10^−1^, 3.49×10^−1^]	[3.08×10^−1^, 3.56×10^−1^]	[3.06×10^−1^, 3.55×10^−1^]
	AIC	**−2.25×10^4^**	**−2.31×10^4^**	**−2.14×10^4^**	**−2.28×10^4^**
	χ^2^ Test	**Pass**			
E2	Point (*ξ_k_*)	3.33×10^−1^	1.43×10^−1^	1.37×10^−1^	8.28×10^−1^
	Interval	[3.59×10^−1^, 7.78×10^−1^]	[1.54×10^−1^, 7.50×10^−1^]	[1.47×10^−1^, 8.14×10^−1^]	[4.18×10^−1^, 8.92×10^−1^]
	AIC	−1.35×10^4^	−1.32×10^4^	−1.34×10^4^	1.59×10^4^
	χ^2^ Test	Fail			
E3	Point (*ξ_k_*)	2.60×10^2^	2.89×10^3^	3.31×10^4^	8.12×10^3^
	Interval	[2.42×10^2^, 2.80×10^2^]	[2.69×10^3^, 3.13×10^3^]	[3.08×10^4^, 3.57×10^4^]	[7.56×10^3^, 8.75×10^3^]
	AIC	−7.55×10^3^	−1.27×10^4^	−1.57×10^4^	−1.82×10^4^
	χ^2^ Test	Fail			

Model E1 was reported by Hunziker, Jensen and Sandeep [37], model E2 is a modified version of E1, and model E3 was suggested by Proctor and Gray [48].

**Figure 5:**
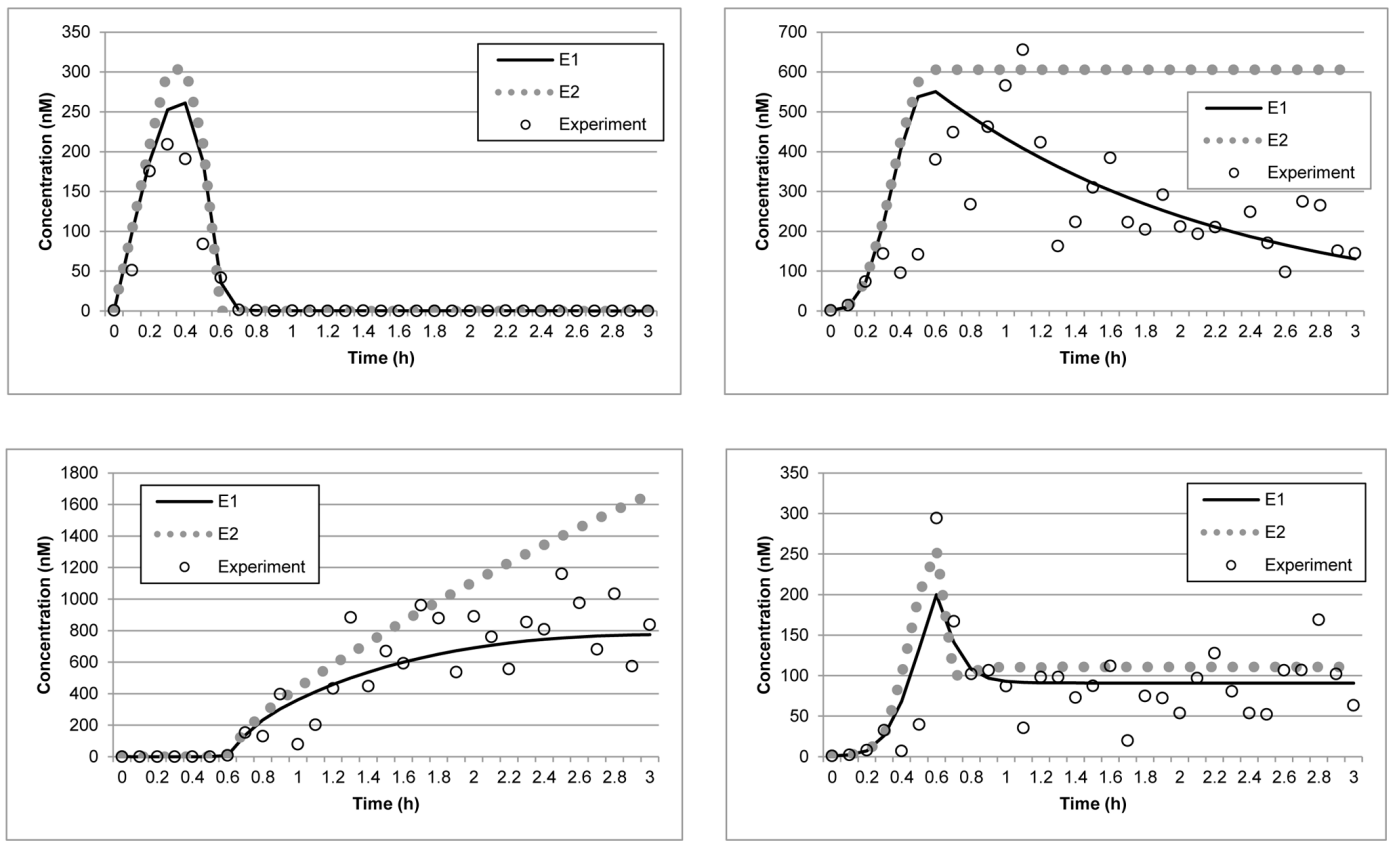
Model selection for the p53 negative feedback loop model. Data points (circles) represent synthetic experimental measurements obtained by adding Gaussian noise to the model prediction. The straight and dash lines represent the reconstructed model, E1, and the modified model, E2, respectively. The X represents the model prediction of the model by Proctor and Gray [48]. The upper left and right panels display the concentrations of nuclear-p53 and Mdm2, respectively. The lower left and right panels show the concentrations of p53-Mdm2 and Mdm2 mRNA, respectively.

Setting *k*_4_ and *k*_9_ to zero perturbs the original model by removing diffusion of the p53-Mdm2 complex into the nucleus and knocking out the Mdm2 gene. Simultaneously, the degradation process of Mdm2 mRNA is also bypassed. This combined perturbation will affect the concentration of each gene product. Table 7 shows that the variance points of each concentration in the new model are not within the expected intervals, indicating that the estimated parameters are inconsistent with the actual dynamics described by the experimental data. Therefore, the estimated parameters can be rejected with 95% confidence level. The AIC results also show that the size of the parameters estimated using the original model is much smaller than those produced by the modified model, further supporting the rejection of the modified model. Figure 5 shows the model selection performed by the proposed method between the reconstructed model, E1, and the modified model, E2.

Using the same experimental data set, we also tested if the method can be used to select the model reported by Hunziker, Jensen and Sandeep [37] over an older model proposed by Proctor and Gray [48] for the p53 signaling pathway negative feedback loop. The Proctor and Gray model, denoted as E3, also specifies the concentrations of nuclear-p53, Mdm2, the p53-Mdm2 complex and Mdm2 mRNA, although the model structure is significantly different. As shown in Figure 5, using the data set, the method was not able to obtain a fit with E3. Table 7 also indicates that E3 fails the χ^2^ test. Overall, the results show that the method can be used to select models based on the experimental data, since only E1 passed the test.

### The Arginine Catabolism Pathway

Arginine is an essential amino acid that has several important roles in mammals, such as wound healing, ammonia removal from the body, and hormone release. Arginine is synthesized from citrulline by the consecutive actions of two cytosolic enzymes, argininosuccinate synthetase and argininosuccinate lyase. The synthesis involves a considerable amount of energy since each molecule of argininosuccinate requires the hydrolysis of adenosine triphosphate (ATP) to adenosine monophosphate (AMP). The amino acid synthesis has also been extensively studied since it is a precursor of nitric oxide, crucial in neurotransmission and immune response [49], [50]. Despite its importance, the dynamic properties of arginine catabolism remain unclear [38].

In this study, the model of the arginine catabolism pathway reported in [38] is used. The model consists of the branch of arginine metabolism leading to either nitric oxide or polyamines in aorta endothelial cells. The following series of equations represent the model [38]:
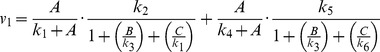
(24)

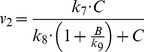
(25)

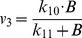
(26)

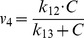
(27)

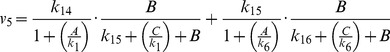
(28)where *A*, *B*, and *C* are external arginine, ornithine, and internal arginine, respectively. In total, the model consists of 16 parameters, but it only considers the concentrations of ornithine and internal arginine, which can be derived from the following ODEs:



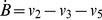
(29)

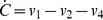
(30)


Here, we present the data fit results of these two concentrations.

**Figure 6:**
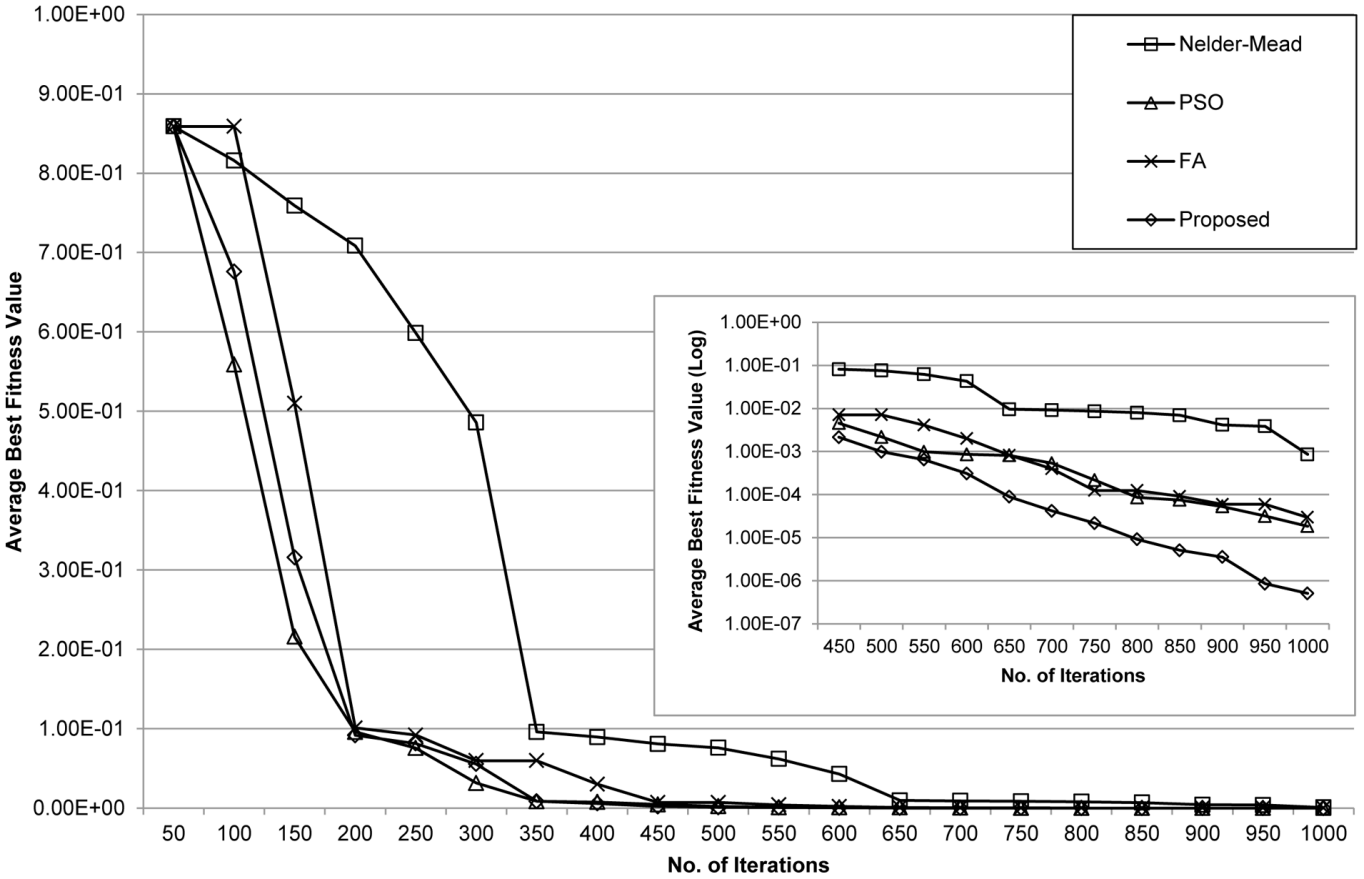
Convergence behaviour for the arginine catabolism model. Plots show the average best fitness values of the Nelder-Mead, PSO, FA and the proposed methods at each iteration.

We evaluated the performance of the proposed method by comparing it with the Nelder-Mead, PSO, and FA methods. The population size, *NP* was set to 25, 50, 75, and 100. Each method was subjected to 100, 500, and 1000 iterations with 100 independent runs. Table 8 lists the resulting average best fitness values. In all cases, the proposed method obtained better fitness values. The convergence behaviour of each method is presented in Figure 6. The results indicate that the proposed method converges faster than the other methods. Additionally, unlike the others, the proposed method can also escape suboptimal solutions. The Nelder-Mead method once again converged to a suboptimal solution. At the beginning of the iterations, the performances of the PSO, FA, and the proposed methods were comparable, which suggests that the three methods are proficient in finding better solutions among the suboptimal solutions. Nonetheless, as shown in Table 9, the proposed method showed faster convergence times than PSO and FA, which is the result of the substantial improvements in the convergence behaviour toward the end of the iterations.

**Table 8:** Average best fitness and standard deviation (presented within bracket) for the arginine catabolism model.

No. of Solutions	No. of Iterations	Nelder-Mead	PSO	FA	Proposed
25	100	1.89×10^−3^ (2.30×10^−3^)	2.11×10^−4^ (1.98×10^−4^)	8.89×10^−5^ (3.35×10^−5^)	9.91×10^−6^ (4.22×10^−6^)
	500	8.94×10^−4^ (5.12×10^−4^)	1.86×10^−4^ (1.03×10^−4^)	2.99×10^−5^ (1.91×10^−5^)	4.10×10^−6^ (3.30×10^−6^)
	1000	5.05×10^−4^ (3.01×10^−4^)	8.19×10^−5^ (2.26×10^−5^)	9.09×10^−6^ (5.61×10^−6^)	8.25×10^−7^ (4.03×10^−7^)
50	100	8.53×10^−4^ (5.56×10^−4^)	9.93×10^−5^ (6.72×10^−5^)	2.51×10^−5^ (2.23×10^−5^)	3.89×10^−6^ (1.02×10^−6^)
	500	3.97×10^−4^ (2.17×10^−4^)	5.01×10^−5^ (3.23×10^−5^)	9.90×10^−6^ (5.13×10^−6^)	8.80×10^−7^ (5.22×10^−7^)
	1000	9.91×10^−5^ (5.57×10^−5^)	9.09×10^−6^ (3.11×10^−6^)	3.87×10^−6^ (1.10×10^−6^)	1.25×10^−7^ (1.02×10^−7^)
75	100	2.52×10^−4^ (2.36×10^−4^)	3.88×10^−5^ (2.01×10^−5^)	9.25×10^−6^ (5.34×10^−6^)	7.73×10^−7^ (3.30×10^−7^)
	500	8.58×10^−5^ (3.20×10^−5^)	9.08×10^−6^ (5.58×10^−6^)	2.95×10^−6^ (1.24×10^−6^)	1.53×10^−7^ (1.19×10^−7^)
	1000	5.35×10^−5^ (2.18×10^−5^)	3.15×10^−6^ (2.05×10^−6^)	9.98×10^−7^ (5.16×10^−7^)	8.55×10^−8^ (1.10×10^−8^)
100	100	1.25×10^−4^ (2.07×10^−4^)	2.23×10^−5^ (1.91×10^−5^)	8.33×10^−6^ (4.24×10^−6^)	5.50×10^−7^ (2.31×10^−7^)
	500	9.98×10^−5^ (3.27×10^−5^)	8.93×10^−6^ (4.59×10^−6^)	5.12×10^−6^ (2.12×10^−6^)	8.53×10^−8^ (2.05×10^−8^)
	1000	6.23×10^−5^ (2.18×10^−5^)	5.38×10^−6^ (2.96×10^−6^)	2.26×10^−6^ (1.95×10^−6^)	2.98×10^−8^ (1.20×10^−8^)

**Table 9:** Average computation times, in second, for the arginine catabolism model. Average times taken for 1000 iterations in 100 independent runs.

No. of Solutions	Average Computation Times (s)			
	Nelder-Mead	PSO	FA	Proposed
25	48.9	55.0	50.9	51.2
50	70.1	85.3	79.6	68.8
75	93.5	106.9	98.1	95.6
100	120.9	136.1	125.5	132.2

**Figure 7:**
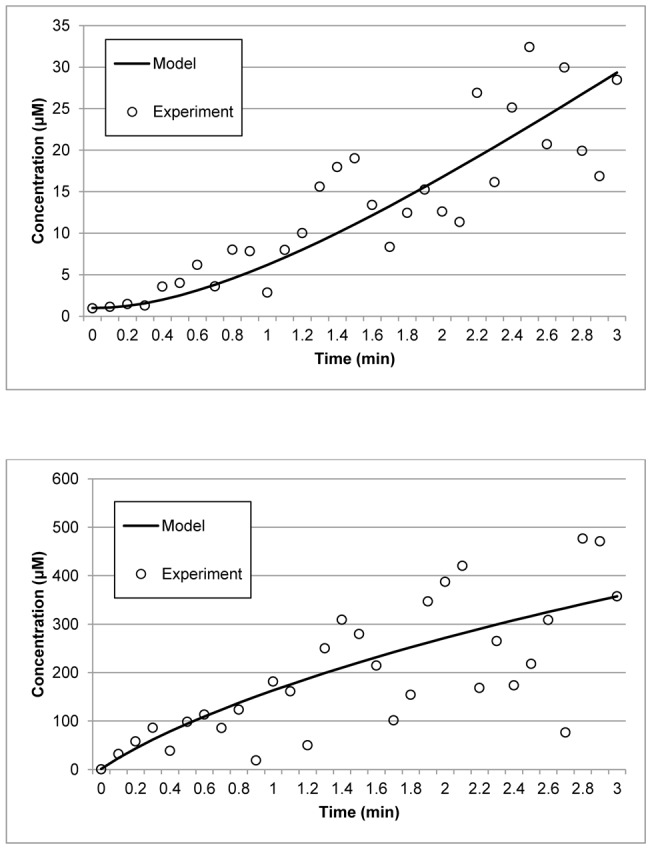
Data fitting for the arginine catabolism model. Data points (circles) represent the synthetic experimental measurements generated by adding the Gaussian noise to the model prediction (crosses). Lines represent the reconstructed model using the parameters estimated by the proposed method. The upper and lower panels represent the concentrations of ornithine and internal arginine, respectively.

We measured the variance of the results from each method to evaluate their reliability. The real variance points were calculated as 3.57×10^−1^ and 3.55×10^−1^ for *B* and *C*, respectively. Tables 10, 11, 12, 13 show the variance points and intervals obtained by the proposed, Nelder-Mead, PSO, and FA methods. The variance points of the proposed method lie within the intervals, and the deviation from the expected points is also very small. However, other methods produced variance points that deviated significantly from the expected values, even though they were still within the intervals. The proposed method is also the only method to pass the χ^2^ test. Figure 7 illustrates a smooth fit of the data generated from the estimated parameters using the proposed method with the data from the experiment. Taken together, these results indicate that the parameters estimated by the proposed method are more reliable.

**Table 10:** χ^2^ test for the parameter estimation of the arginine catabolism model using the proposed method.

	*B*	*C*
Real Variance (*σ_k_^2^*)	3.57×10^−1^	3.55×10^−1^
Variance Point (*ξ_k_*)	3.60×10^−1^	3.55×10^−1^
Variance Interval	[3.32×10^−1^, 3.87×10^−1^]	[3.31×10^−1^, 3.82×10^−1^]
χ^2^ Test	**Pass**	

**Table 11:** χ^2^ test for the parameter estimation of the arginine catabolism model using the Nelder-Mead method.

	*B*	*C*
Real Variance (*σ_k_^2^*)	3.57×10^−1^	3.55×10^−1^
Variance Point (*ξ_k_*)	6.14×10^2^	6.41×10^5^
Variance Interval	[5.58×10^2^, 6.62×10^2^]	[3.64×10^4^, 6.92×10^5^]
χ^2^ Test	Fail	

**Table 12:** χ^2^ test for the parameter estimation of the arginine catabolism model using the PSO method.

	*B*	*C*
Real Variance (*σ_k_^2^*)	3.57×10^−1^	3.55×10^−1^
Variance Point (*ξ_k_*)	1.93×10^1^	6.32×10^5^
Variance Interval	[1.77×10^1^, 2.08×10^1^]	[2.52×10^4^, 6.81×10^5^]
χ^2^ Test	Fail	

**Table 13:** χ^2^ test for the parameter estimation of the arginine catabolism model using the FA method.

	*B*	*C*
Real Variance (*σ_k_^2^*)	3.57×10^−1^	3.55×10^−1^
Variance Point (*ξ_k_*)	8.21×10^2^	2.74×10^4^
Variance Interval	[7.64×10^2^, 1.61×10^3^]	[2.55×10^4^, 2.74×10^4^]
χ^2^ Test	Fail	

Table 14 lists the results of the model selection for the arginine catabolism pathway. The model depicted by Eq. 29–30 is modified by setting the value of parameter *k*_7_ to zero to give a new model:
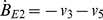
(31)

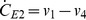
(32)


**Table 14:** Arginine catabolism model selection results.

		*B*	*C*
Real Variance (*σ_k_^2^*)		3.57×10^−1^	3.55×10^−1^
F1	Point (*ξ_k_*)	3.60×10^−1^	3.55×10^−1^
	Interval	[3.32×10^−1^, 3.87×10^−1^]	[3.31×10^−1^, 3.82×10^−1^]
	AIC	**−2.88×10^5^**	**−1.89×10^4^**
	χ^2^ Test	**Pass**	
F2	Point (*ξ_k_*)	6.85×10^−1^	2.66×10^−1^
	Interval	[5.98×10^−1^, 7.38×10^−1^]	[2.87×10^−1^, 6.15×10^−1^]
	AIC	−1.13×10^5^	−9.73×10^3^
	χ^2^ Test	Fail	

Model F1 is a reconstructed model that was reported in [38] and model F2 is a modified version of F1.

**Figure 8:**
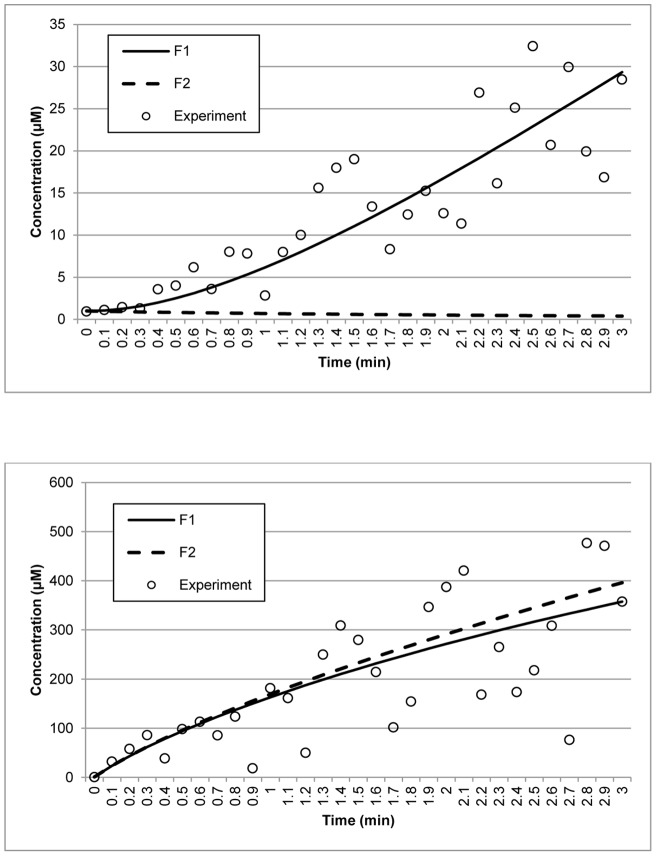
Model Selection for the arginine catabolism model. Data points (circles) represent synthetic experimental measurements obtained by adding Gaussian noise to the model prediction. The straight and dash lines represent the reconstructed model, F1, and the modified model, F2, respectively. The upper and lower panels represent the concentrations of ornithine and internal arginine, respectively.

The model now bypasses the competitive inhibition by ornithine. As listed in Table 14, the variance points of the concentrations in the model differs from the real variance points. More importantly, the variances are not within the intervals given by the model. In addition, the concentrations obtained from the model are rejected by the χ^2^ test. The AIC test also indicates that the variances obtained from the original model are smaller than those of the new model, supporting the decision to reject the latter. Figure 8 shows the selection of the reconstructed model, F1, and the new model, F2, by the proposed method.

**Figure 9:**
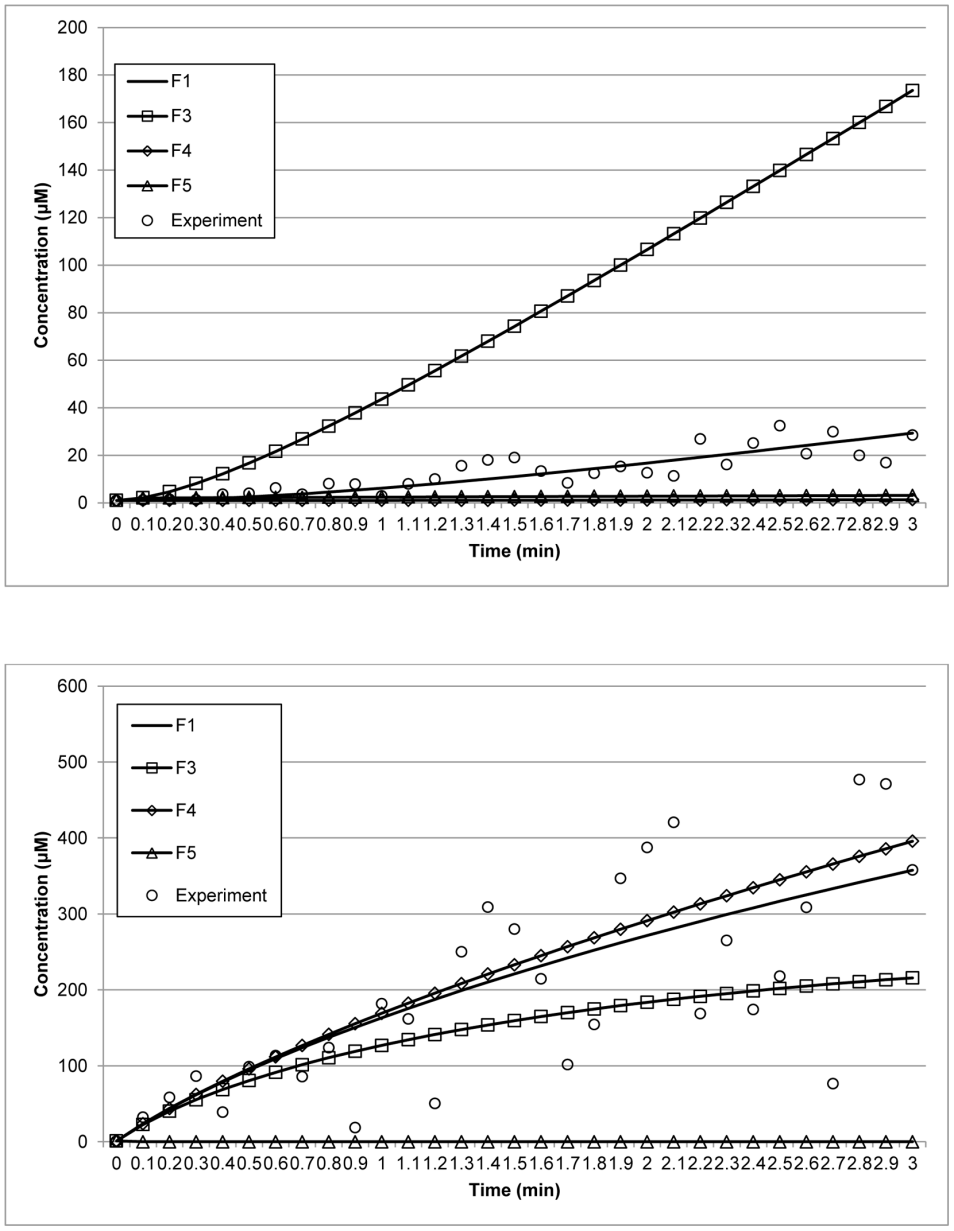
Model Selection for different parameter sets in the arginine catabolism model. Data points (circles) represent synthetic experimental measurements obtained by adding Gaussian noise to the model prediction. The upper and lower panels represent the concentrations of ornithine and internal arginine, respectively.

To further evaluate the robustness of the method, we have also performed model selection tests with different sets of parameter values. Table 15 lists the parameter sets used, which are denoted by F1 (unchanged parameters), F3, F4, and F5, and the corresponding model outputs are given in Figure 9. Table 16 summarizes the results of the χ^2^ test for each parameter set. The results clearly show that F1 is statistically consistent with the selection data, whereas the other parameter sets failed the test. These results indicate that the method is robust to parameter changes and can be used to select the most plausible model for a given data set.

**Table 15:** Evaluated parameter sets of the arginine catabolism model.

Model	Parameters
F1	*k*_1_ = 70, *k*_2_ = 160.5, *k*_3_ = 380, *k*_4_ = 847, *k*_5_ = 420, *k*_6_ = 420, *k*_7_ = 110, *k*_8_ = 1500, *k*_9_ = 1000, *k*_10_ = 0.013, k_11_ = 60, *k*_12_ = 1.33, *k*_13_ = 16, *k*_14_ = 160.5, *k*_15_ = 380, *k*_16_ = 847
F3	*k*_8_ = 100, *k*_15_ = 1, *k*_16_ = 1
F4	*k*_7_ = 1, *k*_10_ = 1, *k*_14_ = 1, *k*_15_ = 1
F5	*k*_2_ = 1, *k*_5_ = 1, *k*_8_ = 1, *k*_15_ = 1, *k*_16_ = 1

**Table 16:** Arginine catabolism model selection results with different parameter sets.

		*B*	*C*
Real Variance (*σ_k_^2^*)		3.53×10^−1^	3.24×10^−1^
F1	Point (*ξ_k_*)	3.52×10^−1^	3.24×10^−1^
	Interval	[3.28×10^−1^, 3.79×10^−1^]	[3.02×10^−1^, 3.49×10^−1^]
	AIC	**−2.25×10^4^**	**−2.31×10^4^**
	χ^2^ Test	**Pass**	
F3	Point (*ξ_k_*)	5.72×10^4^	2.56×10^6^
	Interval	[5.33×10^4^, 6.17×10^4^]	[2.36×10^6^, 2.74×10^6^]
	AIC	−9.21×10^3^	−1.05×10^4^
	χ^2^ Test	Fail	
F4	Point (*ξ_k_*)	6.56×10^4^	1.11×10^6^
	Interval	[6.10×10^4^, 7.10×10^4^]	[1.03×10^6^, 1.20×10^6^]
	AIC	−5.50×10^3^	−1.15×10^4^
	χ^2^ Test	Fail	
F5	Point (*ξ_k_*)	2.21×10^6^	8.50×10^5^
	Interval	[2.06×10^6^, 2.39×10^6^]	[7.88×10^5^, 9.12×10^5^]
	AIC	−4.96×10^3^	−1.29×10^4^
	χ^2^ Test	Fail	

## Discussion

The estimation of parameters is a major issue in the development of accurate and reliable biological models. Models are usually represented with ODEs to simulate the time varying processes that take place within cells. The ODEs depend on parameters that reflect the physiological properties of the system, such as reaction rates and kinetic constants. Since it is difficult to measure all parameters experimentally, the model is predicted by fitting experimental data using nonlinear least square techniques. However, prediction is also a challenge because experimental data are frequently hampered by measurement noise and incompleteness due to experimental limitations. In the past few years, several approaches have been proposed to get around this problem [1], [8], [10], [14], [22]. Especially, evolutionary-based algorithm such as the DE method, has demonstrated to be effective in predicting nonlinear biological models [39], [41] since it can produce robust and reliable estimations [9], [14], [22].

In this paper, we have proposed an optimization method for the parameter estimation and selection of biological models. The method hybridizes the FA and DE approaches. By coupling with an error variance test, the method acquires the reliability of the estimated parameters. Because the variance also determines the selection or rejection of a model, even noisy and incomplete experimental data can be used to estimate the unknown parameters.

Evolutionary algorithms often converge to suboptimal solutions and require a substantial amount of computation time [9], [12]. The proposed method addresses these limitations by improving the neighbourhood search of FA using the random evolutionary search of DE. In one iteration, the solutions obtained from the predicted model are ranked according to the fitness. The ranked population is then classified as potential and weak solutions. The neighbourhood and evolutionarily operations of FA and DE methods are performed to improve the potential solutions, respectively, whereas the weak solutions are randomly repopulated to escape suboptimal solutions. With this improved search, less computation time is needed to find good solutions.

We measure the performance of the method by applying it to two complex and nonlinear biological models: the negative feedback loop of p53 signalling and arginine catabolism. In both cases, the method found better solutions within shorter computation times compared to Nelder-Mead, PSO, or FA approaches. Statistical analysis using error variance and intervals showed that the parameters estimated by the proposed method are reliable and consistent with the experimental data. Hence, the method is able to find reliable and accurate parameters even from noisy and incomplete experimental data. Furthermore, the χ^2^ test showed that the model output generated using the estimated parameters is valid. Strikingly, the Nelder-Mead, PSO, and FA methods all failed this test. The estimated parameters were used for model selection to determine the reliability of the parameters in different experimental conditions. The favorable results of the evaluation demonstrated the consistency of the parameters with the original model and the corresponding experimental data. The results also suggest that the parameters are practically identifiable in different experimental conditions.

Efforts to couple parameter estimation using a hybrid optimization method with statistical analysis to ensure the reliability and accuracy of prediction models have exhibited positive results in recent years [1], [8], [41]. Our work here demonstrates similar outcomes. Since our method can find identifiable parameters from experimental data, it can be employed when designing optimal experiments for parameter estimation [30], [31], [40], [49]. In addition, owing to its reduced computation time, parameters of more detailed nonlinear models such as spatially resolved reaction-diffusion models [51], [52], [53] could also potentially be estimated with the method.
